# Experimental dataset on water levels, sediment depths and wave front celerity values in the study of multiphase shock wave for different initial up- and down-stream conditions

**DOI:** 10.1016/j.dib.2021.107082

**Published:** 2021-04-22

**Authors:** Foad Vosoughi, Mohammad Reza Nikoo, Gholamreza Rakhshandehroo, Amir H. Gandomi

**Affiliations:** a Research Associate, Department of Civil and Environmental Engineering, Shiraz University, Shiraz, Iran; b Associate Professor, Department of Civil and Environmental Engineering, Shiraz University, Shiraz, Iran; c Professor, Department of Civil and Environmental Engineering, Shiraz University, Shiraz, Iran; d Professor, Faculty of Engineering and Information Technology, University of Technology Sydney, Ultimo, NSW 2007, Australia

**Keywords:** Water level data, Sediment depth data, Wave front celerity, Image processing method, Dam-break, Silted-up reservoir, Multiphase shock wave

## Abstract

This data article presents a rich original experimental video sources and wide collections of laboratory data on water levels, sediment depths and wave front celerity values arose from different multiphase dam-break scenarios. The required data of dam-break shock waves in highly silted-up reservoirs with various initial up- and down-stream hydraulic conditions is obtained directly from high-quality videos. The multi-layer shock waves were recorded by three professional cameras mounted along the laboratory channel. The extracted video images were rigorously scrutinized, and the datasets were obtained through the images via image processing method. Different sediment depths in the upstream reservoir and dry- or wet-bed downstream conditions were considered as initial conditions, compromising a total of 32 different scenarios. A total of 198 original experimental videos are made available online in the public repository “Mendeley Data” in 8 groups based on 8 different initial upstream sediment depths [1], [2], [3], [4], [5], [6], [7], [8]. 20 locations along the flume and 15 time snaps after the dam breaks were considered for data collecting. Consequently, a total of 18,000 water level and sediment depth data points were collected to prepare four datasets, which are uploaded in the public repository “Mendeley Data”. A total of 9600 water level data points could be accessed in [9], [10], while 8400 sediment depth data points are available online in [11], [12] and could be utilized for validation and practical purposes by other researchers. This data article is related to another research article entitled “Experimental study and numerical verification of silted-up dam-break” [13].

## Specifications Table

**Table:** 

Subject	Civil and Structural Engineering
Specific subject area	Hydraulic structures and dam engineering
Type of data	Original videos Tables Images Charts
How data were acquired	Measured by means of image processing method using experimental high-quality video images
Data format	Both raw and analyzed
Parameters for data collection	The water levels, sediment depths datasets and wave front celerity values were rigorously measured and classified for different initial conditions including 8 upstream reservoir silting degrees (sediment depths) while the downstream bed was initially dry or wet with 3 standing water depths of 2, 4 and 5 cm which totally constituted 32 distinct dam-break scenarios.
Description of data collection	The water levels and sediment depths data were measured by scrutinizing high-quality video images for 20 locations along the flume and 15 time snaps after the dam breaks. A total of 18,000 data of water levels and sediment depths are presented in this data article. The values of wave front celerity were measured via image processing using extracted video images, firstly for several 1-m-long intervals through downstream channel bed. Then, the mean values of wave front celerity along downstream channel are carefully measured and classified for all dam-break scenarios.
Data source location	Institution: Shiraz University Department: Civil Engineering Department City/Town/Region: Shiraz Country: Iran Latitude and longitude: Hydraulic Lab of Shiraz University, 29.628257, 52.517245 (29°37′41.7″N 52°31′02.1″E)
Data accessibility	Raw images and wave front celerity tables are included in this article. Original videos are uploaded in the public repository [1], [2], [3], [4], [5], [6], [7], [8]. Water level and sediment depth Datasets are available in the public repository [9], [10], [11], [12]. Repository name: Mendeley Data DOI: http://dx.doi.org/10.17632/k74x6vxwxx.3[1] http://dx.doi.org/10.17632/9spmp2fps9.3[2] http://dx.doi.org/10.17632/p6bsdz7xch.3[3] http://dx.doi.org/10.17632/rdbxhtnm5r.3[4]
	http://dx.doi.org/10.17632/dpsxm39y5r.3[5] http://dx.doi.org/10.17632/kktt39fzrv.3[6] http://dx.doi.org/10.17632/pch9mhxr5k.3[7]
	http://dx.doi.org/10.17632/xjcvx48r5f.3[8] http://dx.doi.org/10.17632/nc573y67tp.3[9] http://dx.doi.org/10.17632/zm7rr9ngn5.3[10] http://dx.doi.org/10.17632/fsgc53mgvj.3[11] http://dx.doi.org/10.17632/pr3f5rvj8c.3[12] Direct URL to data: https://data.mendeley.com/datasets/k74x6vxwxx/3[1] https://data.mendeley.com/datasets/9spmp2fps9/3[2] https://data.mendeley.com/datasets/p6bsdz7xch/3[3] https://data.mendeley.com/datasets/rdbxhtnm5r/3[4] https://data.mendeley.com/datasets/dpsxm39y5r/3[5] https://data.mendeley.com/datasets/kktt39fzrv/3[6] https://data.mendeley.com/datasets/pch9mhxr5k/3[7] https://data.mendeley.com/datasets/xjcvx48r5f/3[8] https://data.mendeley.com/datasets/nc573y67tp/3[9] https://data.mendeley.com/datasets/zm7rr9ngn5/3[10] https://data.mendeley.com/datasets/fsgc53mgvj/3[11] https://data.mendeley.com/datasets/pr3f5rvj8c/3[12]
Related research article	F. Vosoughi, G. Rakhshandehroo, M.R. Nikoo, M. Sadegh, Experimental study and numerical verification of silted-up dam break, J. Hydrol. 590 (2020). [13]

## Value of the Data

• These datasets could help to obtain a better scientific comprehension of water levels and sediment depths variation in multiphase shock waves propagation.
• This data article can improve the technical understanding of wave front celerity in dam-break shock flood phenomenon particularly when its reservoir is silted-up.
• The original experimental videos presented in this data article could be utilized for future studies and facilitate reproducibility of wide information in the related article as well.
• The wide obtained datasets could be utilized by other researchers in future studies on silted-up dam break in wet prone areas for validation and practical purposes.
• The literature is sparse on silted-up dam breaks, and no studies have reported or provide datasets concerning water levels, sediment depths and wave front celerity in the case of such phenomenon in wet downstream conditions [14], [15].

## 1 Data Description

In this data article, a large collection of experimental data in investigation of dam break flood waves under different initial upstream sediment depths with dry or wet downstream conditions is provided. The water levels and sediment depths at different locations in the laboratory channel and at various time snaps after the dam break were carefully extracted and presented as well as evaluated rigorously in related research article [13]. The dam break flood wave characteristics in different initial conditions have been well studied, specifically for water-filled reservoirs (without sediment) [16], [17], [18]. This topic is also investigated in the case of dam breaks with dry downstream and high sediment depth in their reservoir, which are called silted-up reservoirs [14], [15]. However, the literature is sparse on silted-up dam breaks, and to the best of the authors’ knowledge, datasets of water level and sediment depth for silted-up dam breaks with wet downstream conditions have not yet to be reported. A set of wave front celerity data of dam-break multiphase flood has been presented in this data article as well. The wave front celerity of dam-break is previously investigated analytically, experimentally and numerically for water-filled reservoir with fix and movable bed condition [18], [19], [20], [21], [22]. Although, this topic is addressed for silted-up reservoirs with dry-bed downstream condition [14], [15], to the best of the authors’ knowledge the data collection of wave front celerity in case of silted-up dam break with wet-bed downstream condition has never been reported to date!

### 1.1 Dam break scenarios

In this data article, the water level, sediment depth and wave front celerity data were measured for different dam break scenarios. Eight distinct reservoir sediment depths, including 0 (no sediment), 3, 7.5, 15, 17.5, 20, 22, and 24 cm, were considered as the upstream initial conditions. The initial level of the reservoir was adjusted to 30 cm in all experiments, hence, the reservoir height is occupied by different upstream sediment depths of 0%−80%. In addition, various initial downstream conditions were considered, including dry- or wet-bed downstream with 3 different standing water levels of 2, 4, and 5 cm. In general, all data collection and videos reported in this data article are related to above-mentioned initial conditions comprised 32 distinct dam-break scenarios, which are listed in Table 1.

**Table 1:** The list of different dam-break scenarios which reported in this article (modified from [13]).

	Initial upstream	Initial downstream
Scenarios no.	sediment depth (cm)	water level (cm)
**1**	0	Dry
**2**	0	2
**3**	0	4
**4**	0	5
**5**	3	Dry
**6**	3	2
**7**	3	4
**8**	3	5
**9**	7.5	Dry
**10**	7.5	2
**11**	7.5	4
**12**	7.5	5
**13**	15	Dry
**14**	15	2
**15**	15	4
**16**	15	5
**17**	17.5	Dry
**18**	17.5	2
**19**	17.5	4
**20**	17.5	5
**21**	20	Dry
**22**	20	2
**23**	20	4
**24**	20	5
**25**	22	Dry
**26**	22	2
**27**	22	4
**28**	22	5
**29**	24	Dry
**30**	24	2
**31**	24	4
**32**	24	5

### 1.2 Water level and sediment depth data

**Fig. 1:**
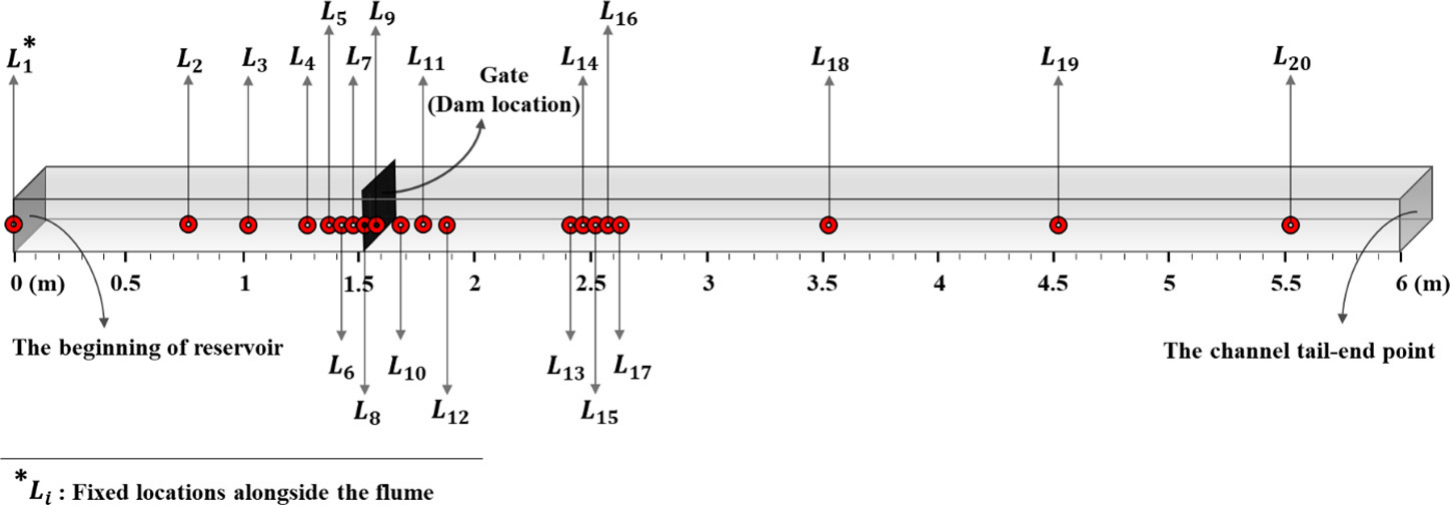
Schematic side view of laboratory flume describing 20 fixed locations alongside the flume, details are not to scale.

The experimental data of water levels and sediment depths were measured and classified according to different locations along the laboratory flume and various time snaps after the dam break. 20 locations along the flume were considered as survey points, where the first location is the reservoir at the starting point of the flume (0.00 cm). The other locations are 76, 102, 127, 137, 142, 147, 152, 157, 167, 177, 187, 242, 247, 252, 257, 262, 352, 452, and 552 cm from the reservoir's beginning (Fig. 1). Gaps between the points near the dam location and 1 m after that were less than other areas along the flume, to ensure enough measurements are performed in this area of high turbulence and rapid depth change and the specific area studied downstream of the dam. The dam section (gate) is located at 152 cm and includes the dam section. 15 screen shots based on the elapsed time after the dam broke were taken at 0.04, 0.08, 0.12, 0.2, 0.3, 0.4, 0.6, 0.8, 1.0, 1.5, 2.0, 3.0, 4.0, 5.0, and 6.0 s from video images for data extraction. Intervals between the snaps were shorter immediately after the dam break and increased gradually.

In general, a total of 18,000 water level and sediment depth data points were obtained then prepared into four datasets, which have been uploaded in the public repository “Mendeley Data” [9], [10], [11], [12]. This data collection includes 9600 water level data and 8400 sediment depth data points, which have been made available as four distinct datasets:

(i) Experimental dataset on **water levels** (*n* = 3600) in studying the influences of dry- and wet-bed downstream conditions on multiphase dam break flood wave, while **0 to 25%** of the dam reservoir is occupied by sedimentation [9].
(ii) Experimental dataset on **water levels** (*n* = 6000) in the investigation of silted-up dam break flood wave for dry- and wet-bed downstream conditions, while **50 to 80%** of the dam reservoir is filled up with sediment [10].
(iii) Experimental dataset on **sediment depths** (*n* = 2400) in analyzing the influences of dry- and wet-bed downstream conditions on multiphase dam break flood wave, while **0 to 25%** of the dam reservoir is occupied by sedimentation [11].
(iv) Experimental dataset on **sediment depths** (*n* = 6000) in the study of silted-up dam break flood wave for dry- and wet-bed downstream conditions, while **50 to 80%** of the dam reservoir is filled up with sediment [12].

Herein, to facilitate the reader's technical understanding and scientific comprehension, three different data tables which presented in [9], [10], [11], [12] are described in detail as an example of the large datasets (60 tables) that were uploaded in the public repository “Mendeley Data” [9], [10], [11], [12]. Table 4 in [9] provides the free surface water level data at 20 different locations along the flume and 15 snap times after the dam break. The initial conditions included the upstream reservoir filled with clear water (no sediment) and a standing water depth of 5 cm in the wet-bed downstream (see Table 4 in [9]). The free surface water level data at all the above-mentioned sections and snap times are presented in Table 6 in [10], while the initial upstream sediment depth was 17.5 cm (58% of the reservoir height), and the downstream was initially wet with a standing water depth of 2 cm (see Table 6 in [10]). Finally, Table 19 in [12] shows the sediment depth data, when the initial upstream sediment depth was 24 cm (80% of the reservoir height), and the initial standing water depth was 4 cm in downstream (see Table 19 in [12]).

**Table 2:** The dam break wave front celerity data for dry initial downstream conditions.

Intervals along the flume (m)	**1.52 - 2.52**	**2.52 - 3.52**	**3.52 - 4.52**	**4.52 - 5.52**
Upstream sediment depth (m)	Measured front wave celerity (m/s)			
0	2.36	2.48	2.28	2.17
0.03	2.30	2.43	2.21	2.11
0.075	2.21	2.33	2.13	2.01
0.15	2.10	2.15	1.99	1.93
0.175	2.05	2.00	1.83	1.79
0.2	1.99	1.82	1.64	1.55
0.22	1.87	1.78	1.59	1.35
0.24	1.60	1.27	1.14	1.09

**Table 3:** The dam break wave front celerity data for wet initial downstream conditions with 2 cm standing water level.

Intervals along the flume (m)	**1.52 - 2.52**	**2.52 - 3.52**	**3.52 - 4.52**	**4.52 - 5.52**
Upstream sediment depth (m)	Measured front wave celerity (m/s)			
0	2.04	2.15	1.84	1.80
0.03	1.90	1.95	1.80	1.74
0.075	1.87	1.90	1.74	1.68
0.15	1.80	1.62	1.59	1.48
0.175	1.75	1.57	1.50	1.40
0.2	1.68	1.47	1.43	1.31
0.22	1.60	1.34	1.27	1.19
0.24	1.38	1.16	1.09	1.08

**Table 4:** The dam break wave front celerity data for wet initial downstream conditions with 4 cm standing water level.

Intervals along the flume (m)	**1.52 - 2.52**	**2.52 - 3.52**	**3.52 - 4.52**	**4.52 - 5.52**
Upstream sediment depth (m)	Measured front wave celerity (m/s)			
0	1.92	2.02	1.74	1.67
0.03	1.85	1.93	1.67	1.59
0.075	1.80	1.87	1.60	1.56
0.15	1.64	1.53	1.50	1.47
0.175	1.60	1.48	1.37	1.32
0.2	1.47	1.37	1.29	1.23
0.22	1.39	1.26	1.17	1.10
0.24	1.26	1.10	1.08	1.08

Pertinent variables of mention include $DW_{L}$ and $S_{d}$, which are the initial downstream water level and initial upstream sediment depth measured in centimeters, respectively. Time refers to the snap times after the sudden removal of the gate in seconds. It should be noted that the vertical column to the left of the tables indicates the distances of different locations (cm) from the beginning point of the laboratory flume. Column *L* indicates all 20 distinct locations along the flume and their distances from the reservoir's beginning in centimeters.

**Fig. 2:**
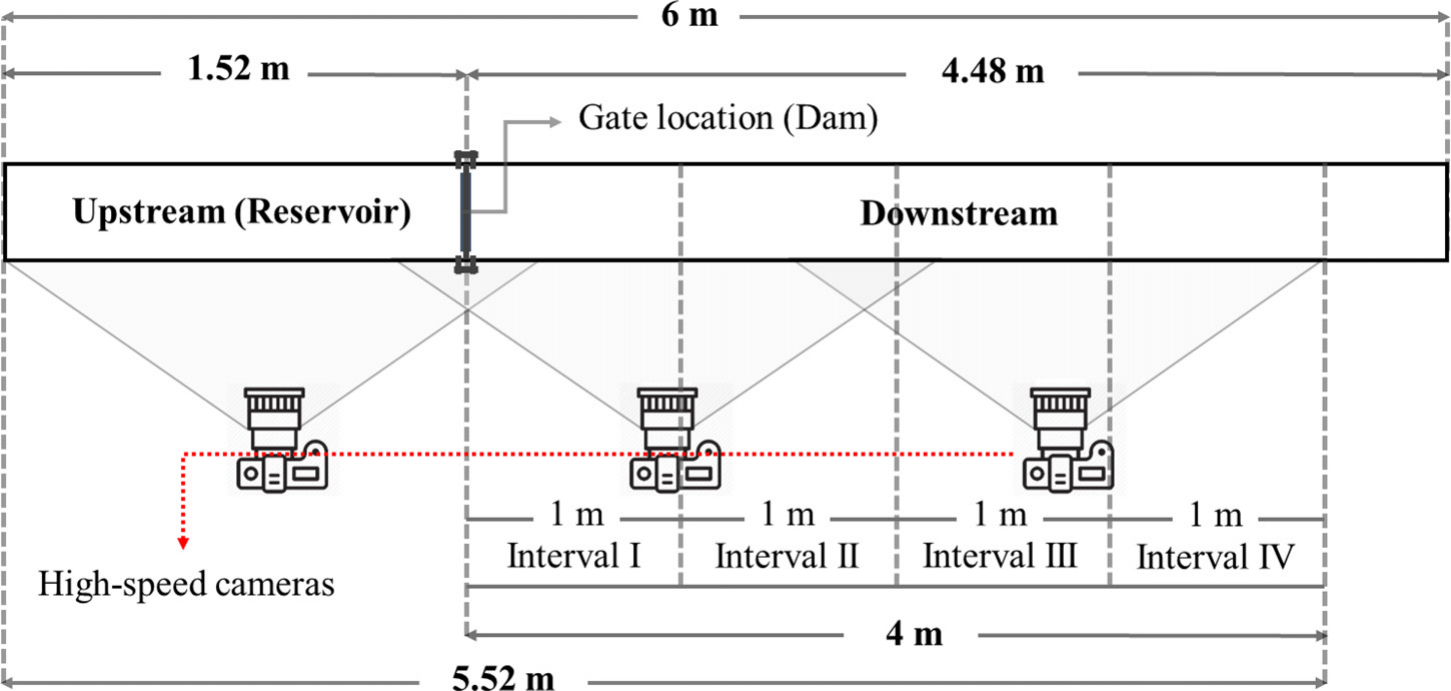
Schematic plan view of laboratory flume, details are not to scale.

**Table 5:** The dam break wave front celerity data for wet initial downstream conditions with 5 cm standing water level.

Intervals along the flume (m)	**1.52 - 2.52**	**2.52 - 3.52**	**3.52 - 4.52**	**4.52 - 5.52**
Upstream sediment depth (m)	Measured front wave celerity (m/s)			
0	1.86	1.95	1.69	1.62
0.03	1.80	1.94	1.62	1.57
0.075	1.75	1.80	1.55	1.49
0.15	1.60	1.50	1.46	1.38
0.175	1.56	1.45	1.36	1.30
0.2	1.44	1.34	1.25	1.20
0.22	1.35	1.23	1.15	1.08
0.24	1.20	1.03	1.05	1.04

**Table 6:** Explanation table of released dam break experiments’ original videos; 1st group [1].

Initial upstream (reservoir) condition: clear water (no sediment)				
Video #	Experiment #	Initial downstream condition	Repetition	Camera #
1	1	Type I 1	1st	1
2	1	Type I	1st	2
3	1	Type I	1st	3
4	2	Type I	2nd	1
5	2	Type I	2nd	2
6	2	Type I	2nd	3
7	3	Type I	3rd	1
8	3	Type I	3rd	2
9	3	Type I	3rd	3
10	4	Type II 2	1st	1
11	4	Type II	1st	2
12	4	Type II	1st	3
13	5	Type II	2nd	1
14	5	Type II	2nd	2
15	5	Type II	2nd	3
16	6	Type III 3	1st	1
17	6	Type III	1st	2
18	6	Type III	1st	3
19	7	Type III	2nd	1
20	7	Type III	2nd	2
21	7	Type III	2nd	3
22	8	Type IV 4	1st	1
23	8	Type IV	1st	2
24	8	Type IV	1st	3
25	9	Type IV	2nd	1
26	9	Type IV	2nd	2
27	9	Type IV	2nd	3

1 Type I: dry-bed.

2 Type II: wet-bed with 2 cm standing water depth.

3 Type III: wet-bed with 4 cm standing water depth.

4 Type IV: wet-bed with 5 cm standing water depth.

**Table 7:** Explanation table of released dam break experiments’ original videos; 2nd group [2].

10% silted-up initial upstream (reservoir) condition: 3 cm sediment depth				
Video #	Experiment #	Initial downstream condition	Repetition	Camera #
28	9	Type I 1	1st	1
29	9	Type I	1st	2
30	9	Type I	1st	3
31	10	Type I	2nd	1
32	10	Type I	2nd	2
33	10	Type I	2nd	3
34	11	Type II 2	1st	1
35	11	Type II	1st	2
36	11	Type II	1st	3
37	13	Type II	2nd	1
38	13	Type II	2nd	2
39	13	Type II	2nd	3
40	14	Type III 3	1st	1
41	14	Type III	1st	2
42	14	Type III	1st	3
43	15	Type III	2nd	1
44	15	Type III	2nd	2
45	15	Type III	2nd	3
46	16	Type IV 4	1st	1
47	16	Type IV	1st	2
48	16	Type IV	1st	3
49	17	Type IV	2nd	1
50	17	Type IV	2nd	2
51	17	Type IV	2nd	3

1 Type I: dry-bed.

2 Type II: wet-bed with 2 cm standing water depth.

3 Type III: wet-bed with 4 cm standing water depth.

4 Type IV: wet-bed with 5 cm standing water depth.

### 1.3 Front wave celerity data

The wave front celerity is firstly calculated in four intervals along the dam downstream which the length of each of them is 1 m. Fig. 2 shows a schematic plan view of the laboratory flume. As it can be seen, these intervals cover 4 m length of the flume in total, from 1.52 m to 5.52 m of the length of the flume. First interval covers from 1.52 m to 2.52 m and 3 other intervals cover 2.52 m to 3.52 m, 3.52 m to 4.52 m and 4.52 m to 5.52 m, respectively. Table 2, presents the wave front celerity values of the silted-up dam break flood in all considered intervals for different initial upstream sediment depths while downstream is initially dry.

**Table 8:** Explanation table of released dam break experiments’ original videos; 3rd group [3].

25% silted-up initial upstream (reservoir) condition: 7.5 cm sediment depth				
Video #	Experiment #	Initial downstream condition	Repetition	Camera #
52	18	Type I 1	1st	1
53	18	Type I	1st	2
54	18	Type I	1st	3
55	19	Type I	2nd	1
56	19	Type I	2nd	2
57	19	Type I	2nd	3
58	20	Type II 2	1st	1
59	20	Type II	1st	2
60	20	Type II	1st	3
61	21	Type II	2nd	1
62	21	Type II	2nd	2
63	21	Type II	2nd	3
64	22	Type III 3	1st	1
65	22	Type III	1st	2
66	22	Type III	1st	3
67	23	Type III	2nd	1
68	23	Type III	2nd	2
69	23	Type III	2nd	3
70	24	Type IV 4	1st	1
71	24	Type IV	1st	2
72	24	Type IV	1st	3
73	25	Type IV	2nd	1
74	25	Type IV	2nd	2
75	25	Type IV	2nd	3

1 Type I: dry-bed.

2 Type II: wet-bed with 2 cm standing water depth.

3 Type III: wet-bed with 4 cm standing water depth.

4 Type IV: wet-bed with 5 cm standing water depth.

**Table 9:** Explanation table of released dam break experiments’ original videos; 4th group [4].

50% silted-up initial upstream (reservoir) condition: 15 cm sediment depth				
Video #	Experiment #	Initial downstream condition	Repetition	Camera #
76	26	Type I 1	1st	1
77	26	Type I	1st	2
78	26	Type I	1st	3
79	27	Type I	2nd	1
80	27	Type I	2nd	2
81	27	Type I	2nd	3
82	28	Type II 2	1st	1
83	28	Type II	1st	2
84	28	Type II	1st	3
85	29	Type II	2nd	1
86	29	Type II	2nd	2
87	29	Type II	2nd	3
88	30	Type III 3	1st	1
89	30	Type III	1st	2
90	30	Type III	1st	3
91	31	Type III	2nd	1
92	31	Type III	2nd	2
93	31	Type III	2nd	3
94	32	Type IV 4	1st	1
95	32	Type IV	1st	2
96	32	Type IV	1st	3
97	33	Type IV	2nd	1
98	33	Type IV	2nd	2
99	33	Type IV	2nd	3

1 Type I: dry-bed.

2 Type II: wet-bed with 2 cm standing water depth.

3 Type III: wet-bed with 4 cm standing water depth.

4 Type IV: wet-bed with 5 cm standing water depth.

**Table 10:** Explanation table of released dam break experiments’ original videos; 5th group [5].

58.3% silted-up initial upstream (reservoir) condition: 17.5 cm sediment depth				
Video #	Experiment #	Initial downstream condition	Repetition	Camera #
100	34	Type I 1	1st	1
101	34	Type I	1st	2
102	34	Type I	1st	3
103	35	Type I	2nd	1
104	35	Type I	2nd	2
105	35	Type I	2nd	3
106	36	Type II 2	1st	1
107	36	Type II	1st	2
108	36	Type II	1st	3
109	37	Type II	2nd	1
110	37	Type II	2nd	2
111	37	Type II	2nd	3
112	38	Type III 3	1st	1
113	38	Type III	1st	2
114	38	Type III	1st	3
115	39	Type III	2nd	1
116	39	Type III	2nd	2
117	39	Type III	2nd	3
118	40	Type IV 4	1st	1
119	40	Type IV	1st	2
120	40	Type IV	1st	3
121	41	Type IV	2nd	1
122	41	Type IV	2nd	2
123	41	Type IV	2nd	3

1 Type I: dry-bed.

2 Type II: wet-bed with 2 cm standing water depth.

3 Type III: wet-bed with 4 cm standing water depth.

4 Type IV: wet-bed with 5 cm standing water depth.

**Table 11:** Explanation table of released dam break experiments’ original videos; 6th group [6].

67.7% silted-up initial upstream (reservoir) condition: 20 cm sediment depth				
Video #	Experiment #	Initial downstream condition	Repetition	Camera #
124	42	Type I 1	1st	1
125	42	Type I	1st	2
126	42	Type I	1st	3
127	43	Type I	2nd	1
128	43	Type I	2nd	2
129	43	Type I	2nd	3
130	44	Type II 2	1st	1
131	44	Type II	1st	2
132	44	Type II	1st	3
133	45	Type II	2nd	1
134	45	Type II	2nd	2
135	45	Type II	2nd	3
136	46	Type III 3	1st	1
137	46	Type III	1st	2
138	46	Type III	1st	3
139	47	Type III	2nd	1
140	47	Type III	2nd	2
141	47	Type III	2nd	3
142	48	Type IV 4	1st	1
143	48	Type IV	1st	2
144	48	Type IV	1st	3
145	49	Type IV	2nd	1
146	49	Type IV	2nd	2
147	49	Type IV	2nd	3

1 Type I: dry-bed.

2 Type II: wet-bed with 2 cm standing water depth.

3 Type III: wet-bed with 4 cm standing water depth.

4 Type IV: wet-bed with 5 cm standing water depth.

**Table 12:** Explanation table of released dam break experiments’ original videos; 7th group [7].

73.3% silted-up initial upstream (reservoir) condition: 22 cm sediment depth				
Video #	Experiment #	Initial downstream condition	Repetition	Camera #
148	50	Type I 1	1st	1
149	50	Type I	1st	2
150	50	Type I	1st	3
151	51	Type I	2nd	1
152	51	Type I	2nd	2
153	51	Type I	2nd	3
154	52	Type II 2	1st	1
155	52	Type II	1st	2
156	52	Type II	1st	3
157	53	Type II	2nd	1
158	53	Type II	2nd	2
159	53	Type II	2nd	3
160	54	Type III 3	1st	1
161	54	Type III	1st	2
162	54	Type III	1st	3
163	55	Type III	2nd	1
164	55	Type III	2nd	2
165	55	Type III	2nd	3
166	56	Type IV 4	1st	1
167	56	Type IV	1st	2
168	56	Type IV	1st	3
169	57	Type IV	2nd	1
170	57	Type IV	2nd	2
171	57	Type IV	2nd	3

1 Type I: dry-bed.

2 Type II: wet-bed with 2 cm standing water depth.

3 Type III: wet-bed with 4 cm standing water depth.

4 Type IV: wet-bed with 5 cm standing water depth.

**Table 13:** Explanation table of released dam break experiments’ original videos; 8th group [8].

80% silted-up initial upstream (reservoir) condition: 24 cm sediment depth				
Video #	Experiment #	Initial downstream condition	Repetition	Camera #
172	58	Type I 1	1st	1
173	58	Type I	1st	2
174	58	Type I	1st	3
175	59	Type I	2nd	1
176	59	Type I	2nd	2
177	59	Type I	2nd	3
178	60	Type II 2	1st	1
179	60	Type II	1st	2
180	60	Type II	1st	3
181	61	Type II	2nd	1
182	61	Type II	2nd	2
183	61	Type II	2nd	3
184	62	Type II	3rd	1
185	62	Type II	3rd	2
186	62	Type II	3rd	3
187	63	Type III 3	1st	1
188	63	Type III	1st	2
189	63	Type III	1st	3
190	64	Type III	2nd	1
191	64	Type III	2nd	2
192	64	Type III	2nd	3
193	65	Type IV 4	1st	1
194	65	Type IV	1st	2
195	65	Type IV	1st	3
196	66	Type IV	2nd	1
197	66	Type IV	2nd	2
198	66	Type IV	2nd	3

1 Type I: dry-bed.

2 Type II: wet-bed with 2 cm standing water depth.

3 Type III: wet-bed with 4 cm standing water depth.

4 Type IV: wet-bed with 5 cm standing water depth.

Table 3, provides the wave front celerity values of dam break flood in four above-mentioned intervals along dam downstream for different initial upstream sediment depths while downstream is initially wet with 2 cm standing water. The wave front celerity values of dam break flood in specified intervals, for different initial upstream sediment depths while downstream bed is initially wet with 4 cm standing water are detailed in Table 4.

Finally, Table 5, details the wave front celerity data of dam break flood in all downstream intervals for different initial upstream sediment depths while downstream bed is initially wet with 5 cm standing water.

It worth mentioning that the mean values of wave front celerity through downstream part of the flume for all 32 scenarios of dam-break experiment are measured and presented in related original paper (see Table 3 in [13]). The variance values related to measured mean wave front celerity data along the flume downstream were calculated and detailed there, as well [13].

### 1.4 Original experimental videos

Herein, a rich collection of original videos are made available online in the public repository “Mendeley Data” might be utilized for validation purposes in future studies. The presented video files are related to dam break multiphase flood shock wave experiments which performed in the Shiraz University, Civil and Environmental Engineering Department's Hydraulic Lab (Shiraz, Iran). Considering three cameras which covered length of the flume, 4 different dam break scenarios and 2 or 3 repetitions conducted for each test, a total of 198 videos collected and presented in this document. The experimental videos are classified in 8 groups based on 8 different initial upstream sediment depths [1], [2], [3], [4], [5], [6], [7], [8]. The additional explanations related to video files can be seen in explanation tables of videos (Tables 6-13).

**Fig. 3:**
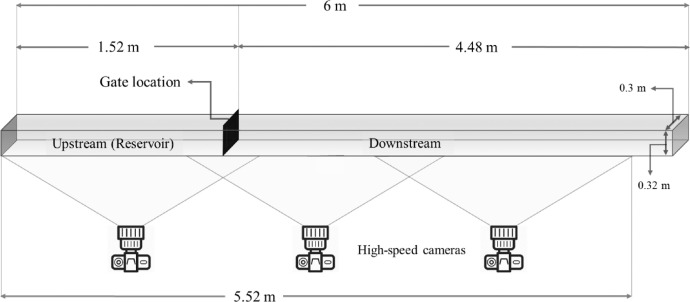
Schematic plan view of laboratory flume; details are not to scale [13].

**Fig. 4:**
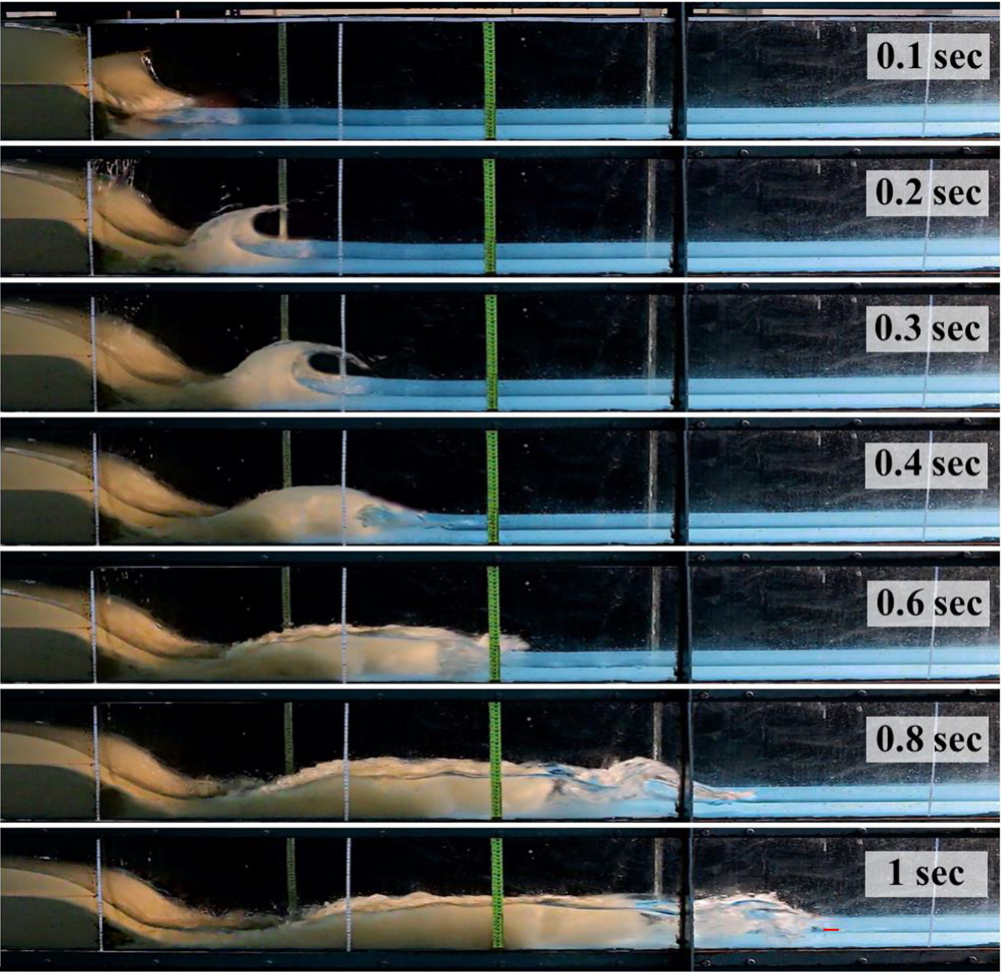
Seven snapshots of video images recorded by second camera related to seven time snaps after the dam break. Initial upstream sediment depth = 15 cm. Initial downstream water level = 4 cm.

The list of 27 video files associated with dam-break experiments when their reservoirs were filled by clear water (no sediment) are detailed in Table 6. Different initial downstream condition types also investigated including dry-bed downstream and wet-bed downstream with different levels of standing water; 2 cm, 4 cm and 5 cm [1]. Table 7, shows the list of 24 videos associated with dam break experiments when the initial upstream sediment depth was 3 cm which makes the upstream reservoir 10% silted-up with respect to the total 30 cm height of the reservoir for different initial downstream conditions [2]. The list of videos related to dam break experiments when the initial upstream sediment depths were 7.5, 15, 17.5, 20, 22 and 24 cm (20% to 80% silted-up reservoirs) for different initial downstream conditions are detailed in Tables 8-13, respectively [3], [4], [5], [6], [7], [8].

**Fig. 5:**
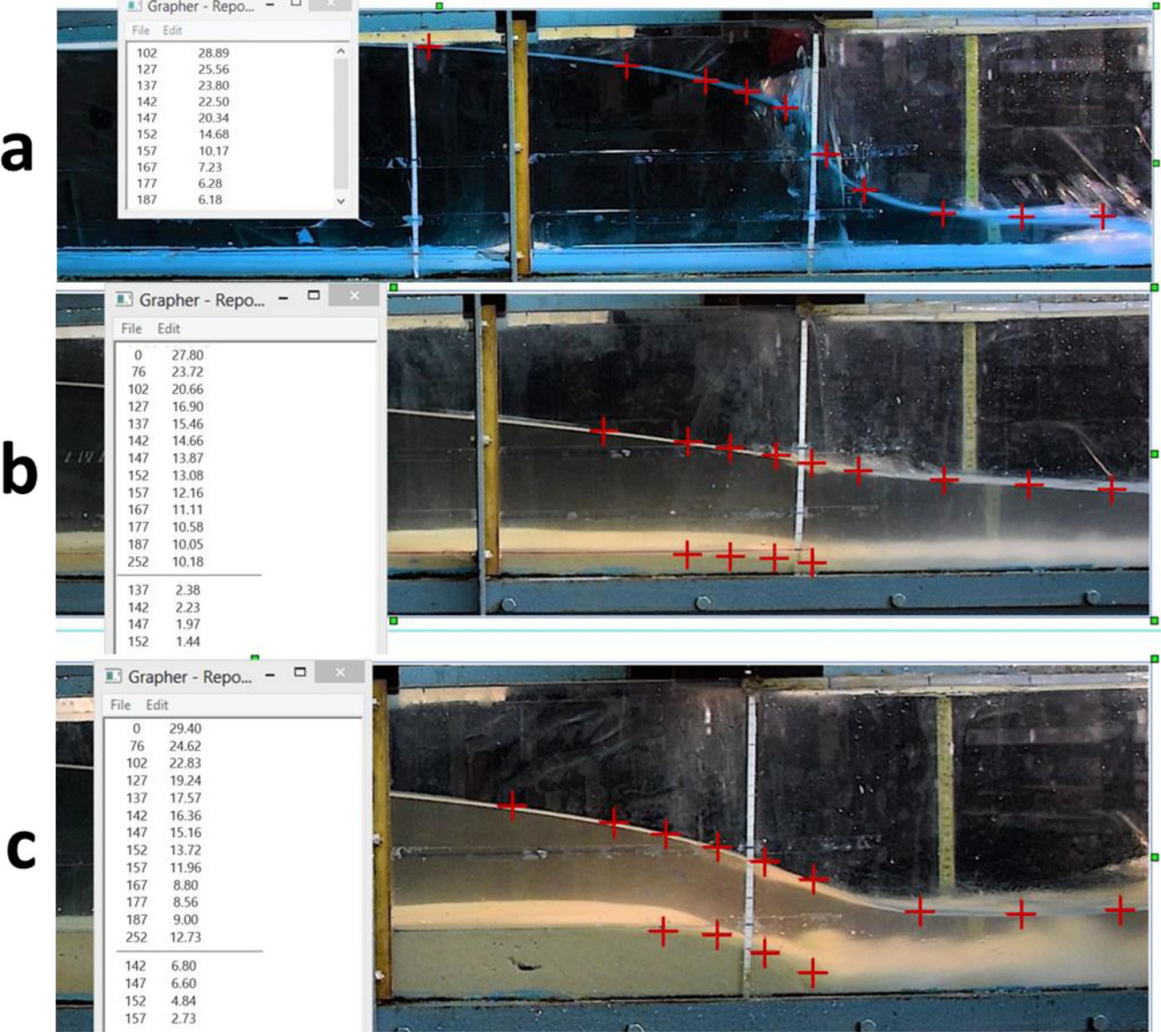
Extracting water level and sediment depth data using Grapher, (a) clear water upstream and dry downstream at 0.2 s, (b) 3 cm sediment depth in upstream and 2 cm standing water in downstream at 1 s, (c) 7.5 cm sediment depth in upstream and 4 cm standing water in downstream at 1 s.

**Fig. 6:**
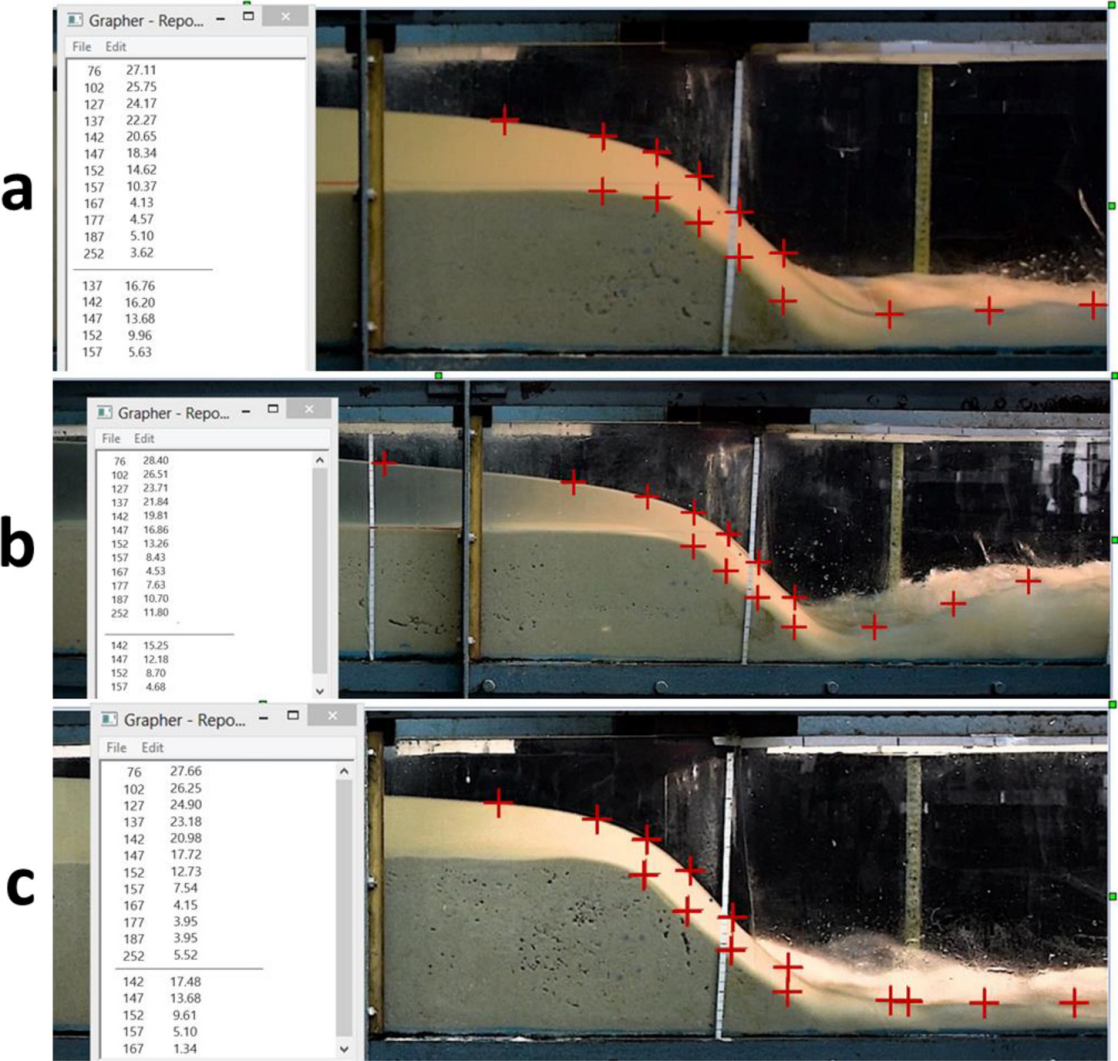
Extracting water level and sediment depth data at 1 s after the dam break using Grapher, (a) 17.5 cm sediment depth in upstream and dry downstream, (b) 17.5 cm sediment depth in upstream and 5 cm standing water in downstream, (c) 20 cm sediment depth in upstream and dry downstream.

## 2 Experimental Design, Materials and Methods

The experimental dam break tests were conducted in the Hydraulic Lab of Civil and Environmental Engineering Department at Shiraz University, Shiraz, Iran. Three high-speed professional cameras mounted along the channel were used to record 50 frames per second (Canon EOS 70D). Each camera covered 2 m over the length of the flume, where considering overlap areas the total distance covered by the cameras was 5.52 m (Fig. 3).

Fig. 4 presents the set of high quality video images recorded by the second camera, depicting the multiphase shock flood wave alongside the laboratory flume at different time snaps after the failure of the dam. The initial upstream sediment depth was 15 cm, while the wet downstream contained 4 cm standing water as the initial downstream condition (Fig. 4). Seven snapshots taken at 0.1, 0.2, 0.3, 0.4, 0.6, 0.8, and 1.0 s associated with different times after the dam broke were classified.

For data extraction, a non-intrusive technique was applied to each of the video images using Grapher® software. In this method, after specifying the coordinates of two certified points on a diagonal line within each of the images, coordinates of any arbitrary point in the x and y axes can be easily obtained by clicking on that point (Figs. 5 and 6). Hence, the free surface water level and sediment depth profiles for any arbitrary time after the dam break could be collected directly from the video images at any location along the laboratory channel, without disturbing the flow with any physical instrument. This method can yield high-quality and accurate data despite being laborious and time-consuming. All video images collectively comprised a total of 18,000 data points, which are available in “Mendeley Data” [9], [10], [11], [12].

Fig. 5(a-c) display the software interior area in extracting water level and sediment depth data. Fig. 5(a) contains a snapshot of the shock flood wave at 0.2 s after the dam break when the upstream reservoir was filled with clear water (no sediment), and the downstream bed was dry. Fig 5(b) presents a snapshot at 1 s after the dam break when the initial upstream sediment depth was 3 cm, and the downstream channel bed was initially wet with 2 cm of standing water. Fig. 5(c) contains a snapshot of the multiphase flood wave at 1 s after the dam break, while the initial upstream sediment depth was 7.5 cm and the initial downstream water level was 4 cm.

Fig. 6(a-c) shows the software interior area in extracting water level and sediment depth data. Fig. 6(a) displays a snapshot of the shock flood wave at 1 s after the dam break when the initial upstream sediment depth was 17.5 cm and the downstream bed was dry. Fig. 6(b) presents a snapshot at 1 s after the dam break with an initial upstream sediment depth of 17.5 cm and wet downstream channel bed with 5 cm standing water. Fig. 6(c) contains a snapshot of the multiphase flood wave at 1 s after the dam break when the initial upstream sediment depth was 20 cm with dry-bed downstream conditions.

The values of wave front celerity were calculated using extracted video images firstly at four intervals along the dam downstream (Fig. 2). Then, the mean values of wave front celerity through dam downstream are carefully measured and classified for all dam break scenarios. To calculate the wave front celerity Eq. (1) was used.

(1) $$C_{f}=\frac{L_{2}-L_{1}}{t_{2}-t_{1}}\;$$

where, *C_f_* represents wave front celerity in (*m/s*), *L_i_* is the distances from reservoir beginning in (*m*) and *t_i_* is the time snap after the dam breaks in (*sec*).

**Fig. 7:**
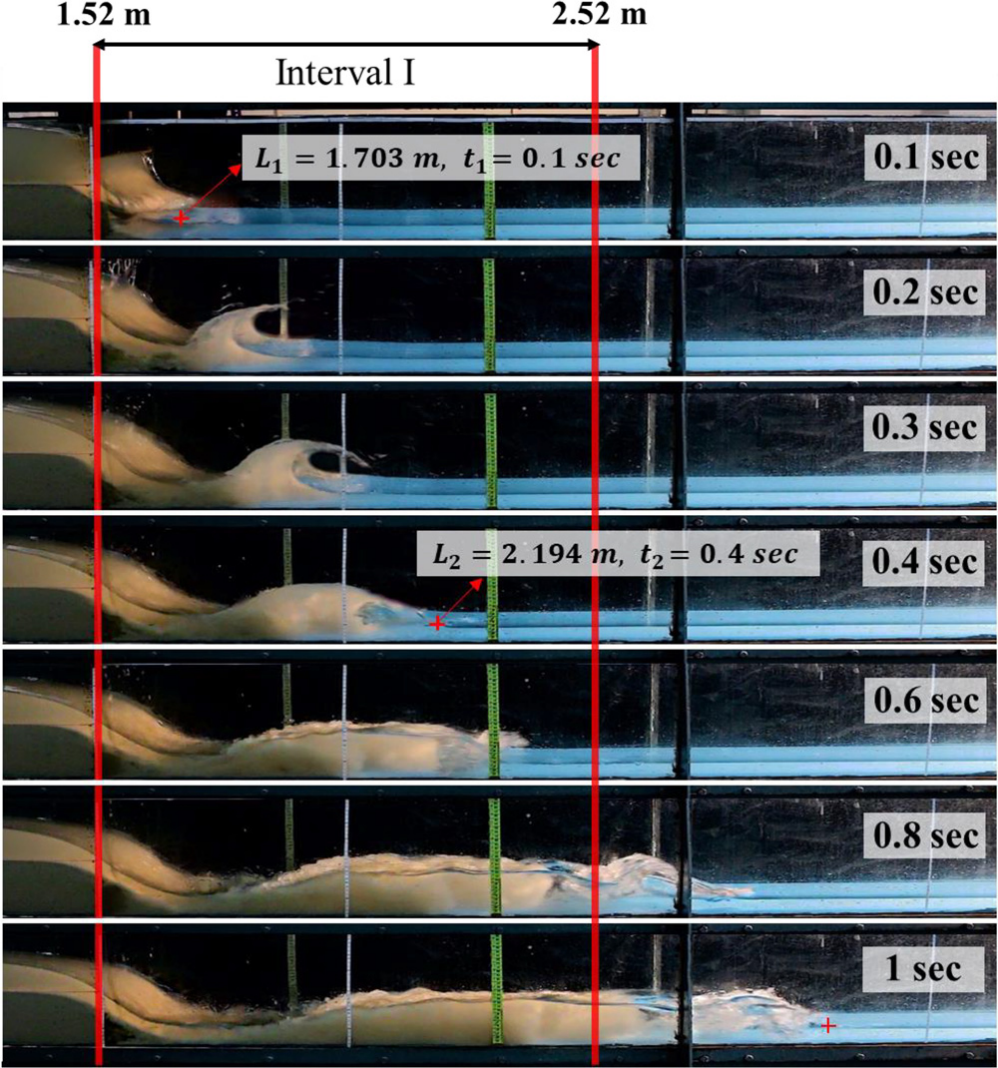
Silted-up dam-break wave advancing at the dam downstream, upstream initial sediment depth is 15 cm, dam downstream bed was initially wet with 4 cm standing water. Red lines indicate boundaries of the first interval.

Fig. 7, shows video images related to fifteenth dam break scenario (Table 1) in which the initial upstream sediment depth was 15 cm and dam downstream bed was initially wet using 4 cm of standing water. As it can be seen, seven-time snaps of 0.1, 0.2, 0.3, 0.4, 0.6, 0.8 and 1 s after the failure of the dam are presented in the figure and the first interval is specified between two vertical red lines. Considering *L_1_, L_2_, t_1_* and *t_2_* the wave front celerity is 1.6367 (m/s), as presented in Table 4.

This process is conducted for all 32 scenarios and 4 intervals and classified in Tables 2 to 5 and then the mean values of wave front celerity through downstream channel are carefully measured for all experiment scenarios (see Table 3 in [13]).

## Statement of Consent and Authorization

The authors state that they have received informed consent from the individuals in question to publish all videos.

The individuals were freely and voluntarily consent to take part in the research study and they authorized the use and disclosure of their information in connection with the study. Those individuals have gave their explicit written consent and received a signed copy of the consent and authorization form.

Each individual who was appeared in any video has made aware in advance of the fact that such videos are being taken and of all the purposes for which they might be used, including publication in Data in Brief.

Written consents will be retained by the author and copies of the consents will be provided to the Data in Brief Editors upon request.

## CRediT Author Statement

**Foad Vosoughi:** Conceptualization, Methodology, Validation, Investigation, Resources, Data Curation, Writing - Original Draft, Visualization, Writing - Review & Editing; **Mohammad Reza Nikoo:** Conceptualization, Methodology, Supervision, Writing - Review & Editing; **Gholamreza Rakhshandehroo:** Conceptualization, Methodology, Supervision, Writing - Review & Editing; **Amir H. Gandomi:** Conceptualization, Supervision, Writing - Review & Editing.

## Declaration of Competing Interest

The authors declare that they have no known competing financial interests or personal relationships that have, or could be perceived to have, influenced the work reported in this article.
